# Complications of exercise and pharmacologic stress echocardiography

**DOI:** 10.3389/fcvm.2023.1228613

**Published:** 2023-08-03

**Authors:** Christopher Lee, Sam Dow, Kajal Shah, Stanislav Henkin, Cynthia Taub

**Affiliations:** Heart and Vascular Center, Dartmouth-Hitchcock Medical Center, Dartmouth College, Lebanon, NH, United States

**Keywords:** stress echocardiogram, stress test complications, stress test contraindications, exercise stress, dobutamine, vasodilator, coronary artery disease

## Abstract

Stress echocardiography is a diagnostic cardiovascular exam that is commonly utilized for multiple indications, including but not limited to the assessment of obstructive coronary artery disease, valvular disease, obstructive hypertrophic cardiomyopathy, and diastolic function. Stress echocardiography can be performed via both exercise and pharmacologic modalities. Exercise stress is performed with either treadmill or bicycle-based exercise. Pharmacologic stress is performed via either dobutamine or vasodilator-mediated (i.e., dipyridamole, adenosine) stress testing. Each of these modalities is associated with a low overall prevalence of major, life-threatening adverse outcomes, though adverse events are most common with dobutamine stress echocardiography. In light of the recent COVID-19 pandemic, the risk of infectious complications to both the patient and stress personnel cannot be negated; however, when certain precautions are taken, the risk of infectious complications appears minimal. In this article, we review each of the stress echocardiographic modalities, examine major potential adverse outcomes and contraindications, assess the risks of stress testing in the setting of a global pandemic, and examine the utilization and safety of stress testing in special patient populations (i.e., language barriers, pediatric patients, pregnancy).

## Introduction

Stress echocardiography is a widely accessible and cost-effective stress testing modality that has been utilized for the past 45 years (1). Of an estimated 8.7 million patients who annually undergo non-invasive diagnostic testing for obstructive coronary artery disease in the United States, approximately 31% undergo stress echocardiography (2). Stress echocardiography is performed via multiple modalities and includes exercise stress testing, either via a treadmill or upright/supine bicycle, or pharmacologically-mediated testing with the administration of dobutamine or vasodilators (i.e., dipyridamole or adenosine) (3). Both exercise and pharmacologic stress echocardiography have excellent sensitivities and specificities in the diagnosis of obstructive coronary artery disease, with a sensitivity of between 80% and 85% for exercise stress echocardiography and 79%–83% for dobutamine stress echocardiography, along with a specificity between 80% and 88% and 82% to 85%, respectively (3, 4). Since the introduction of stress echocardiography, the indications have expanded to not only include an assessment of coronary artery disease, but to also encompass indications such as an assessment of valvular disease (i.e., mitral stenosis, aortic stenosis), congenital heart disease, obstructive hypertrophic cardiomyopathy, diastolic function, and myocardial viability. The preferred modality of stress echocardiography differs based on the indication. With the increasing utilization of stress echocardiography, stress protocols have become incrementally more aggressive and have been utilized for more complex, higher-risk patients (5).

With any test that is routinely and widely performed, the safety and potential complications are particularly important for both clinical and medicolegal purposes. Prior data from a large registry of 85,997 patients undergoing stress echocardiography found that life-threatening events only occurred in 86 (∼0.1%) of all tests (6). The safety of the personnel conducting these tests is also of particular importance, especially in light of the coronavirus pandemic (7). This review will summarize the complications and adverse effects of exercise and pharmacologic stress echocardiography to both patients and stress personnel and review contraindications for each modality. We also review the role and safety of stress echocardiography in valvular disease and special patient populations.

## Exercise stress echocardiography

Exercise-based stress testing is the modality of choice if a patient is able to exercise (American Society of Echocardiography Class I Recommendation, Level of Evidence A) (3). With exercise, functional status can be gauged, cardiac electromechanical response is preserved, and symptoms, or lack thereof, can be linked with activity (3, 8). The normal physiologic response to exercise involves an increase in full body oxygen demand, though primarily from working muscles, with a subsequent increase in cardiac output. There is an increased chronotropic and inotropic cardiovascular response, leading to a reduction in systemic vascular resistance to optimize oxygen delivery to the working muscle groups (9).

Both treadmill and bicycle exercise can be employed with exercise stress testing. The Bruce protocol is most frequently utilized for treadmill protocols with image acquisition at baseline and immediately following peak exertion (10). With bicycle exercise, the initial protocol begins at a workload of 25 watts, increasing by increments of 25 watts every 2–3 min, and can be accomplished either upright or supine (3). Diagnostic endpoints include a heart rate goal of ≥85% of maximum predicted heart rate (MPHR), limiting severe chest pain or anginal equivalent, or positivity via electrocardiographic (ECG) criteria. In patients with limited mobility, alternative exercise protocols that have been utilized include upper extremity maneuvers via handgrip exercise (11–14) or via cycle ergometer (15–17). Though data is limited, static active leg lifts may be considered as an alternative exercise modality as well (18). Passive leg raising may be utilized for preload augmentation to enhance the identification of patients with heart failure with preserved ejection fraction (19, 20).

### Patient complications

The overall risk of major complications with exercise stress echocardiography is exceedingly low. Multiple studies have assessed the risk of complications with an exercise-based protocol. One study surveyed 71 centers from 17 countries, finding that of a total of 26,295 patients undergoing exercise echocardiography, a life-threatening event occurred in only 1 in every 6,574 patients (0.015%) (6). There were a total of 4 life-threatening complications reported (1 acute myocardial infarction [AMI], 2 sustained ventricular tachycardia [VT], 1 cardiac rupture). A multi-center European study collected data from 29 Portuguese and Spanish hospitals, reporting that among 10,975 treadmill exercise echocardiograms, only 4 significant complications occurred (0.03%) with 2 arrhythmias [ventricular fibrillation (VF) and sustained VT], 1 AMI, and 1 ventricular rupture (21). A single-center Spanish study published similar findings—of 19,239 treadmill exercise echocardiograms performed over a 21-year period, the risk of complications was approximately 0.02% to 0.04% (22). The most frequent complications were attributable to arrhythmias (sustained and non-sustained VT, supraventricular tachycardia, atrial fibrillation), atrioventricular block (complete heart block, 2:1 block), and AMI. Other studies have reported similar results (23–25) (Table 1).

**Table 1:** Exercise and pharmacologic stress echocardiographic complications and contraindications.

	Exercise	Dobutamine	Vasodilator
Risk of Major Complications	∼0.015% to 0.04%	∼0.2%	∼0.08%
Most Common Major Complications	Sustained VT, VF, AMI	Sustained VT, VF, AMI	AMI, asystole, hypotension/shock
Other Major Complications	High grade/complete heart block, atrial fibrillation, supraventricular tachycardia	Death, cardiac rupture, CVA, asystole, atrial fibrillation/flutter, supraventricular tachycardia, high grade/complete heart block	Bradycardia/high grade/complete heart block, CVA, VF, sustained VT
Contraindications	Active ACS<48 h, persistent and poorly controlled ventricular or supraventricular tachyarrhythmias, malignant hypertension, symptomatic, severe aortic valve stenosis, acute aortic dissection, severe, poorly controlled heart failure, acute pulmonary embolism, severe, obstructive LVOT obstruction, acute pericarditis and/or myocarditis		
Other Specific Contraindications	Impaired mobility, active infectious and communicable diseases (i.e., COVID-19, tuberculosis), severe pulmonary hypertension, severe COPD	Significant history of ventricular arrhythmias; atropine should be avoided in patients with narrow-angle glaucoma, pyloric stenosis, or myasthenia gravis	Active wheezing or severe reactive airway disease (i.e., COPD, asthma), severe hypotension (systolic blood pressure <90 mmHg), known high grade atrioventricular block without a functional pacemaker,
Infectious Risk	Though there is evidence of increased viral transmissibility of active infectious and communicable diseases when heart rate is at ≥50% (with exercise) of the maximal predicted heart rate, if patients undergo pre-test screening and appropriate health care personnel precautions are taken, there is a minimal risk of transmission to healthcare personnel.		

ACS, Acute coronary syndrome; AMI, Acute myocardial infarction; COPD, Chronic obstructive pulmonary disease; CVA, Cerebrovascular accident; LVOT, Left ventricular outflow tract; VF, Ventricular fibrillation; VT, Ventricular tachycardia.

In terms of exercise machine safety, treadmill-based stress testing, as compared to bicycle-based exercise, is associated with an elevated risk of physical injury. Emergency department-reported injuries due to mechanical home exercise equipment is disproportionately comprised of injuries due to treadmills, which pose an inherent risk of patient falls (26). Treadmill-based exercise may also be associated with more arrhythmic major adverse events, such as premature ventricular contractions (PVCs) and VT, as compared to bicycle-based exercise (27).

### Viral transmission risks to patients and healthcare personnel

Throughout the COVID-19 pandemic, echocardiography laboratories drastically reduced the number of patients undergoing stress echocardiography to minimize the risk of viral transmission. Internationally, at the height of the pandemic, there was an 83% reduction in the number of stress echocardiograms that were performed (28). Much of the focus has been on the potential for increased viral transmissibility to both patients and healthcare personnel. With exercise, there is an augmented risk of aerosol generation, which is increased to statistically higher concentrations when exercise is performed at ≥50% of the predicted heart rate (7, 29). Highly infectious COVID-19 variants, such as omicron, heightened the concern of viral transmissibility (30). However when exercise stress testing is performed with certain precautions, such as pre-test screening—either by antigen testing or screening for symptoms, assessing local community rates of infection, ensuring use of personal protective equipment (PPE), and maintaining appropriate room ventilation, the risk of viral transmissibility appears to be minimal. A single center study in the United Kingdom that prospectively collected data on 740 consecutive patients undergoing exercise stress echocardiography at the height of the COVID-19 pandemic in 2020 found that when healthcare personnel wore adequate PPE, patients wore surgical masks covering both their nose and mouth, and there was adequate ventilation in the stress echocardiography laboratory, the viral transmission risk to healthcare personnel was minimal (31). Patient face mask utilization, though leading to increased exertional dyspnea, does not appear to significantly impact patient functional capacity, the results of exercise stress testing, or lead to adverse patient outcomes (32).

### Contraindications

Though generally well-tolerated, there are contraindications to exercise stress echocardiography that should be followed to minimize adverse events. Contraindications include patients with active acute coronary syndrome (ACS) <48 h, persistent and poorly controlled ventricular or supraventricular tachyarrhythmias, malignant hypertension, symptomatic severe aortic valve stenosis, acute aortic dissection, severe, symptomatic, poorly controlled heart failure, acute pulmonary embolism, acute pericarditis and/or myocarditis, and severe obstructive left ventricular outflow tract (LVOT) obstruction (3, 4, 33, 34). Importantly, even patients without a resting LVOT obstruction may develop exertional hypotension and/or syncope due to an inducible, latent LVOT obstruction (35). Patients with impaired mobility, inability to exercise, and other co-existing conditions, such as severe pulmonary hypertension or severe chronic obstructive pulmonary disease (COPD), should be considered for an alternative stress testing modality. Patients with active infectious and communicable diseases (i.e., COVID-19, tuberculosis), should have stress testing postponed until adequate treatment of the underlying disease-process and recovery to minimize the risk to both the patient and health personnel (36). Of note, the presence of a left bundle branch block (LBBB) reduces the diagnostic specificity of exercise-based stress testing to detect obstructive coronary artery disease. There is a high false positive rate with the utilization of exercise stress echocardiography due to both a decreased left ventricular contractile response in a large proportion of patients and difficulty in interpreting septal wall motion (37). Additionally, exercise nuclear stress perfusion imaging with single-photon emission computed tomography (SPECT) leads to a high false positive rate due to the presence of reversible septal perfusion defects (38). Exercise-based stress testing in the LBBB population may lead to increased unnecessary invasive testing.

## Pharmacologic stress echocardiography

Pharmacologic stress echocardiography is performed in patients who are unable to exercise or in whom the indication for stress testing requires a pharmacologic modality (i.e., low flow, low gradient aortic stenosis). Pharmacologic agents such as dobutamine, dipyridamole, and adenosine, have been utilized (3). Dobutamine selectively acts on the beta-1 and beta-2 receptors, leading to increased myocardial contractility and smooth muscle dilatation (39). Protocols utilizing dobutamine infusions are initiated at doses of 5 or 10 ug/kg/min, increasing every 3 min to a maximum dose of 40 ug/kg/min. If the target heart rate goal (≥85% of MPHR) is not achieved at a maximum dobutamine infusion, atropine is administered at 0.25 mg at 1 min intervals (up to 1–2 mg). At low doses, dobutamine exerts a predominantly positive inotropic effect, while at higher doses, dobutamine exhibits a chronotropic effect as well (3). Other protocols have been proposed, including accelerated, continuous dosing of dobutamine (40–44), extending the maximum amount of time patients receive dobutamine at 40 ug/kg/min (i.e., from 3 min to 6 min) instead of utilizing atropine (45), and increased maximum doses of dobutamine (i.e., up to 50 ug/kg/min) (46) though no clear advantages with these protocols have been demonstrated.

Vasodilatory agents, such as dipyridamole and adenosine, are utilized as well. Dipyridamole is a phosphodiesterase and adenosine deaminase inhibitor, functioning both as an antiplatelet agent and a coronary vasodilatory agent (47, 48). By inhibiting the breakdown of adenosine, intrinsic levels of adenosine increase and interact with adenosine receptors in a non-selective manner. The targeted receptor is the A_2A_ receptor, which leads to coronary vasodilation and increased coronary flow. A_2A_ receptor activation can also lead to peripheral vasodilation. However, non-specific activation can occur of the A_1_ and A_2B_ receptors. A_1_ receptor activation can lead to bradycardia and atrioventricular conduction abnormalities; A_2B_ receptor activation can lead to bronchoconstriction and peripheral vasodilation (49). Dipyridamole stress protocols infuse a total of up to 0.84 mg/kg of dipyridamole over 10 min and may also incorporate the use of atropine. Adenosine-based stress protocols infuse up to a total dose of 140 ug/kg/min of adenosine over 6 min (34). Regadenoson-based stress echocardiography protocols are emerging as well (50). Regadenoson is a selective A_2A_ receptor agonist that exhibits decreased reactivity with the A_1_ and A_2B_ receptors (51). It is the first selective A_2A_ receptor agonist to be approved by the Food and Drug Administration and has been approved since April 2008 for radionuclide myocardial perfusion imaging. Regadenoson-based myocardial perfusion protocols utilize a single, bolus weight-unadjusted dose of 0.4 mg. Though both adrenergic pharmacologic stress testing with dobutamine and vasodilatory pharmacologic stress testing with dipyridamole are widely utilized, regional preferences for a pharmacologic strategy do exist. Vasodilatory agents are widely used in Europe as compared to the United States, where adrenergic stress testing is more widely utilized (52).

### Patient complications

The risk of major complications with all pharmacologic stress echocardiography testing modalities is considerably low, though of the agents that are utilized, the risk of major complications is highest with dobutamine. With dobutamine-based protocols, due to its catecholaminergic effects, the most common life-threatening complications are ventricular tachyarrhythmias and AMI. The incidence of VF is ∼0.04%, sustained VT is ∼0.15%, and AMI is ∼0.02% (53). Other major complications include death, cardiac rupture, cerebrovascular accident (CVA), and cardiac asystole (<0.01%). A multicenter, international registry study of 71 centers by Varga et al. found that the risk of life-threatening adverse events in 35,103 total patients undergoing dobutamine stress echocardiography was roughly 1 in every 557 patients (0.18%, 63 total) (6). The most common complications were sustained VT (*n* = 27), VF (*n* = 11), and AMI (*n* = 11), followed by cardiac rupture (*n* = 5), CVA (*n* = 3), asystole (*n* = 2), complete heart block (*n* = 2), and hypotension/shock (*n* = 2). In addition to the risk of ventricular arrhythmias, there is an elevated risk of atrial arrhythmias (i.e., atrial fibrillation, atrial flutter, supraventricular tachycardia) (53, 54). Another European multi-center study found that of 6,832 patients who underwent dobutamine stress echocardiography, there were 26 total major complications (10 sustained VT, 3 VF, 6 AMI, 2 cardiac ruptures, 1 CVA, 1 death, 2 complete heart block, and 1 severe hypotension) (21). Single center studies have reported similar results with minor variations—finding that overall, the risk of life-threatening complications with dobutamine stress echocardiography was low, though when encountered, the most common complications were ventricular tachyarrhythmias (VT, VF) and AMI (55–62) (Table 1).

Mechanistically, dobutamine may induce both sustained and non-sustained VT for multiple reasons: effects on action potential, QRS, and QTc interval duration, increased intracellular calcium leading to increased ventricular automaticity, reduction in plasma potassium levels, and myocardial ischemia (53). VF appears to mostly occur in patients with structural heart disease along with severe myocardial ischemia; acute myocardial infarction may be induced by increased shear forces leading to the disruption of unstable plaque. Dobutamine-induced platelet activation may also lead to coronary vessel occlusion (63).

Like dobutamine stress echocardiography, the risk of serious adverse events with vasodilator-based stress testing is also very low. Varga et al. reported a risk of major, life-threatening complications with dipyridamole echocardiographic stress testing of only 1 in every 1,294 patients (0.08%) (6). Of 24,599 patients, there were a total of 19 major adverse events (5 AMI, 4 asystole, 4 hypotension/shock, 3 CVA, 2 VF, and 1 sustained VT). A multicenter registry study by Picano et al. reported similar results of 10,451 patients undergoing dipyridamole stress echocardiography (64). There were a total of 7 major adverse reactions (0.07%)—3 AMI, 2 cardiac asystole, 1 sustained VT, and 1 pulmonary edema. Other significant adverse effects that were reported included symptomatic hypotension, bradycardia, second degree 2:1 atrioventricular block, complete heart block, supraventricular tachycardia, and atrial fibrillation (Table 1).

Despite the favorable safety risk profile of vasodilator stress testing, vasodilators are associated with a high prevalence of minor side effects, which is seen in more than 80% of cases (65). Most commonly, hypotension and/or bradycardia, headache, dizziness, dyspnea, chest pain, bronchoconstriction, and nausea/vomiting may be present. Mechanistically, most of the increased minor side effect profile appears to likely be due to receptor cross-reactivity. Bradycardia and high-grade heart block are likely mediated by A_1_ receptor activation. Hypotension due to peripheral vasodilation is mediated by both A_2A_ and A_2B_ receptor activation (49), which in turn can also induce tachyarrhythmias via a baroreflex activation or through increased sympathetic tone. Bronchoconstriction is associated with A_2B_ receptor activation.

### Contraindications

Though pharmacologic stress echocardiography shares similar contraindications with exercise stress echocardiography, certain additional contraindications should be considered. Due to pharmacologic vasodilator cross-reactivity with the A_1_ and A_2B_ receptors, vasodilator stress testing should be avoided in patients with active wheezing or severe reactive airway disease (i.e., COPD, asthma) due to the risk of worsening bronchospasm or severe hypotension (systolic blood pressure <90 mmHg) due to peripheral vasodilation (3). Vasodilator pharmacologic stress testing should also be avoided in patients with known second degree atrioventricular block, Mobitz II, or complete heart block without a functional pacemaker, due to cross-reactivity with the A_1_ receptor, which can lead to worsening atrioventricular conduction abnormalities. On the other hand, dobutamine is associated with more arrhythmic complications than vasodilator stress testing and should be avoided in patients with a significant history of ventricular arrhythmias (34). Additionally, the use of atropine, which is an anticholinergic agent, as an adjunctive agent to dobutamine is contraindicated in patients with narrow-angle glaucoma, pyloric stenosis, or myasthenia gravis (53).

### Dobutamine stress testing in atrial fibrillation

Dobutamine stress echocardiography in patients with atrial fibrillation has been associated with unpredictable responses in heart rate, along with difficulty in interpreting wall motion abnormalities due to the irregularity of ventricular depolarization. With exercise or dobutamine infusion, increased atrioventricular conduction velocity often leads to the achievement of heart rate goals at lower levels of exercise or dobutamine infusion (66); this may be associated with a lower cardiac workload and subsequent decreased sensitivity in detecting obstructive coronary artery disease. Though few studies have assessed the accuracy and long-term prognostic value of dobutamine stress echocardiography in patients with permanent atrial fibrillation, these studies reported similar levels of diagnostic accuracy for patients with atrial fibrillation as compared to patients in sinus rhythm (67, 68). Dobutamine stress testing in patients with atrial fibrillation, though, may be associated with an increased incidence of arrhythmic complications, most notably wide QRS complex tachycardias. Though patients with atrial fibrillation can undergo a dobutamine-based stress echocardiogram with relative preservation of sensitivity and diagnostic accuracy for obstructive coronary artery disease, physicians and healthcare personnel conducting the tests should be aware of increased arrhythmic complications in this patient population.

### Stress testing in valvular heart disease

Stress echocardiography is also utilized for diagnosing the etiology and severity of valvular lesions. Dobutamine stress echocardiography is utilized to decipher between severe and pseudo-severe aortic stenosis in low flow, low gradient aortic stenosis with reduced ejection fraction. Exercise-based stress testing and in some situations, dobutamine stress echocardiography, can be used to delineate exercise capacity, symptoms, and doppler parameters for patients with rheumatic mitral stenosis and mitral regurgitation.

In patients with low flow, low gradient aortic stenosis with reduced ejection fraction [aortic valve area (AVA) ≤1 cm^2^ with resting maximum velocity <4 m/s, left ventricular ejection fraction <50%, and stroke volume index ≤35 ml/m^2^], low dose dobutamine stress echocardiography is recommended to help decipher between severe and pseudo-severe aortic stenosis [American College of Cardiology/American Heart Association (ACC/AHA) Class IIa recommendation] (69, 70). The protocol for low dose dobutamine stress testing differs from that for coronary indications, beginning with a dobutamine dose of 2.5–5 mcg/kg/min, with increases in dose of 2.5–5 mcg/kg/min every 3–5 min. The maximum dose of dobutamine is 20 mcg/kg/min (71). The dobutamine infusion is stopped when reaching either the maximum dose of dobutamine, obtaining a positive result (defined as a change in stroke volume of >20% from baseline, an increase in aortic jet velocity ≥4.0 m/s, or the achievement of a mean aortic gradient of >30–40 mmHg), exceeding a heart rate of >100 bpm or 10–20 bpm over the baseline heart rate, or with symptoms, a fall in blood pressure, or arrhythmic complications.

Similarly, exercise stress echocardiography may be utilized in asymptomatic patients with severe chronic, primary mitral regurgitation to assess for exertional symptoms and to obtain hemodynamic data by doppler echocardiography (ACC/AHA Class IIa recommendation) (69). Exercise-induced symptoms can also reasonably be assessed in patients with at least moderate mitral regurgitation. Poor prognostic indicators with exercise stress include any increase in the severity of mitral regurgitation (≥1 grade), an increase in systolic pulmonary artery pressure to ≥60 mmHg, lack of left ventricular contractile reserve (left ventricular ejection fraction increases by <5% or global longitudinal strain changes by <2%), or load-dependent right ventricular dysfunction (72). In addition to primary mitral regurgitation, exercise or pharmacologic stress testing is also recommended in patients with chronic, secondary mitral regurgitation as well to assess for an ischemic etiology of mitral regurgitation and to determine myocardial viability (ACC/AHA Class I recommendation) (69). Exercise stress testing can also unmask and/or quantify symptoms in this patient population and pulmonary artery pressures with stress should be measured, as pulmonary artery hypertension is associated with poor outcomes (73). However, patients with ischemic mitral regurgitation with viable myocardium may have improved longer term valvular outcomes (74, 75).

There is also a role for stress testing in patients with rheumatic mitral stenosis. This patient population should undergo stress testing when there is a discrepancy between patient symptoms and the resting echocardiographic findings. In asymptomatic patients with severe mitral stenosis on the resting echocardiogram (mitral valve area ≤1.5 cm^2^ and a diastolic pressure half-time ≥150 ms) or in symptomatic patients with non-severe mitral stenosis, an exercise stress echocardiogram can be utilized to assess for symptoms and assess valvular and pulmonary hemodynamics (ACC/AHA Class I recommendation) (69, 70, 72). Exercise-based stress echocardiography is preferred in this patient population as symptoms can be correlated to exercise capacity and valvular hemodynamics. An increase in the mean mitral gradient to >15 mmHg with exertion is considered significant (72). An exertional systolic pulmonary artery pressure of >60 mmHg is also considered hemodynamically significant for mitral stenosis. Alternatively, in patients who are unable to exercise, dobutamine-based stress echocardiography can be performed, though this stress testing modality is considered inferior to exercise-based stress testing as functional capacity and exercise-induced symptoms cannot be quantified (70). When quantified via dobutamine stress echocardiography, an increase in the mean mitral gradient to >18 mmHg with exertion is considered significant and may portend a poor prognosis without mitral valve intervention (76).

### Patient complications

There are comparatively few studies assessing the safety of performing stress testing in patients with severe valvular dysfunction as compared to patients with intermediate-risk obstructive coronary artery disease. A single center study by Bermejo et al. found that of 35 patients who underwent low dose dobutamine stress testing, 4 patients (11%) had angina, 1 patient (3%) developed atrial tachycardia, and 1 patient (3%) became hypotensive with dobutamine (77). Another single center study by Bountioukos et al. reported that of 20 total patients with severely reduced left ventricular ejection fraction (mean of 25%) undergoing low dose dobutamine stress testing, 4 patients (20%) developed non-sustained VT. Two other single center studies found minimal adverse effects: one study of 24 patients found adverse events in only 1 of 24 patients (4%; non-sustained VT) and the other study of 50 patients reported no ventricular tachyarrhythmias, but did report atrial flutter (2%), hypotension (2%), and chest pain/dyspnea (6%). Though low dose dobutamine stress echocardiography appears generally safe to perform in patients with low flow, low gradient aortic stenosis, it may be associated with a higher risk of arrhythmic complications (particularly ventricular tachyarrhythmias) along with angina/dyspnea and hypotension.

Similar to low flow, low gradient aortic stenosis, data regarding the safety of stress testing in patients with mitral valvular dysfunction is also limited, though stress testing in mitral stenosis appears to be well-tolerated. A single center study of 48 patients with rheumatic mitral valve disease undergoing exercise stress echocardiography by Lev et al. reported no significant adverse events in any patients (78). A larger study by Gentry et al. of 515 patents undergoing exercise treadmill stress testing for suspected significant mitral stenosis (all etiologies, only 33% rheumatic) also reported no significant adverse events with exercise—no malignant arrhythmias, blood pressure abnormalities, or deaths (79). Reis et al. reported data from 37 patients with rheumatic mitral stenosis who underwent dobutamine stress echocardiography, finding that further dobutamine infusion was precluded by arrhythmias—not specified further (4%), hypotension (4%), and dizziness (4%). Overall, patients undergoing stress echocardiography for valvular disease represent a complex, higher-risk group of patients for which the frequency of major adverse events during exercise and pharmacologic stress testing have not been well-delineated and requires closer monitoring.

## Special patient populations

### Hypertrophic cardiomyopathy

Hypertrophic cardiomyopathy (HCM) is a genetic cardiac disorder leading to left ventricular hypertrophy, myocyte fibrosis, and myocyte disarray. HCM is an important cause of arrhythmias, sudden cardiac death, and heart failure (80). In this population, patients with LVOT obstruction have an increased likelihood of functional limitations and an elevated risk of mortality, with a worsened mortality trend associated with increasing LVOT obstruction (81, 82). Unfortunately, LVOT gradients are often dynamic and may not be detected in up to half of resting echocardiograms (even with provocative maneuvers, such as Valsalva or squat-to-stand) (83). Exercise stress echocardiography is frequently utilized in this population to elucidate exertional symptoms that may be attributable to LVOT obstruction. In symptomatic patients with a resting LVOT gradient of <50 mmHg on transthoracic echocardiogram, exercise stress echocardiography is recommended to induce a significant LVOT gradient of ≥50 mmHg [ACC/AHA and European Society of Cardiology (ESC) Class I recommendation] and, in asymptomatic patients, exercise stress echocardiography carries an ACC/AHA Class IIa and ESC Class IIb recommendation (83, 84).

In patients with HCM, stress testing is often performed for multiple reasons but include as an assessment for an inducible LVOT gradient, functional capacity, and obstructive coronary artery disease. Exercise stress echocardiography is the test of choice when stress testing is performed to assess LVOT gradients and functional capacity and has been shown to be safe to perform. Drinko et al. performed consecutive exercise stress echocardiograms in 263 patients with HCM with a mean LVOT gradient at rest of 38 mmHg and a stress peak LVOT gradient of 74 mmHg (85). Patients were continued on their medications prior to stress testing. Only 1 patient (0.4%) experienced a major complication (hemodynamically stable, non-sustained VT requiring cardioversion). Minor complications were common, with up to 12.9% of patients developing presyncope, 11.7% with chest pain, 4.2% with non-sustained ventricular arrhythmias, and 3% with non-sustained atrial arrhythmias. Bunch et al. similarly assessed the safety of performing exercise stress testing in HCM patients (mean resting LVOT gradient of 17 mmHg that increased to 56 mmHg with stress) (86). Major complications only occurred in 1 patient (1.2%) with hemodynamically stable, non-sustained VT. Minor complications were common with arrhythmias occurring in 45% of patients (33% premature ventricular contractions, 27% premature atrial contractions, 2% atrial fibrillation). Other exercise echocardiographic and clinical parameters that may serve as markers of functional and clinical deterioration in the HCM population include worsened left atrial contractile strain (87, 88), right ventricular function (i.e., global longitudinal strain, tricuspid annular plane systolic excursion) (89), and pulmonary congestion (i.e., B-lines on lung ultrasound) (90). As compared to exercise stress testing, pharmacologic modalities to assess an inducible LVOT gradient are limited. Dobutamine lacks specificity to determine an inducible LVOT obstruction as it can provoke LVOT gradients even in patients without HCM and has a high rate of false positives (83, 91), can decrease coronary filling time (92, 93), and should not be utilized to determine therapeutic treatment options for a LVOT obstruction. Another strategy to assess severe latent LVOT obstruction on resting echocardiography than via provocative resting maneuvers includes amyl nitrite inhalation. Amyl nitrite is a vasodilatory agent that can be utilized to identify a severe inducible LVOT gradient. Amyl nitrite has been shown to more frequently provoke a latent LVOT obstruction than Valsalva, and nearly as robustly as exercise stress testing (94).

In contrast, there is a limited role for stress testing for the assessment of obstructive coronary artery disease in the HCM population. The prevalence of myocardial ischemia, as detected on stress myocardial perfusion imaging, has been shown to exceed that of the prevalence of obstructive coronary artery disease (95). Myocardial ischemia, in this population, is multifactorial and may be due to obstructive coronary artery disease, LVOT obstruction, microvascular dysfunction, arrhythmias, decreased coronary artery vasodilatory reserve, inadequate capillary density in hypertrophied myocardium, or myocardial bridging (96). Echocardiography-based stress modalities may be limited by abnormal wall motion of the hypertrophic myocardium and the use of dobutamine, as a pharmacologic modality, may provoke a LVOT gradient and is not recommended (97). Other pharmacologic agents, such as vasodilator agents, have a high prevalence of false positive results in the detection of myocardial ischemia due to obstructive coronary artery disease (95). Given the lack of specificity posed by stress testing modalities in the HCM population, in patients with a moderate to high pre-test probability of obstructive coronary artery disease, a coronary angiogram is the preferred modality to rule out obstructive coronary artery disease (98).

### Language barriers

Language barriers are common across the United States and patients with limited English proficiency make up between 19 and 21 million patients nationwide (99). Patients with limited English proficiency and other language barriers receive an overall lower level of care due to their language barrier, though the use of professional interpreters raises this quality of care to approach that of patients without language barriers (100, 101). In addition to evaluating patients in their non-primary language, the use of untrained interpreters can affect diagnostic accuracy and subsequent clinical care (102). As compared to professional interpreters, untrained *ad hoc* interpreters make significantly higher numbers of errors of potential consequence (103, 104). One cross-sectional study of 583 patients found that patients with limited English proficiency had 2.8-fold higher odds of not reporting a history of cardiovascular disease as compared to patients without limited English proficiency (105). This is of particular concern in patients who are undergoing stress testing and in whom the presence or progression of symptoms is of particular importance. In order to minimalize adverse events and poor patient outcomes, patients with limited English proficiency should have a professional interpreter available, either in person, by phone, or via video conference call, during the entirety of stress testing. However, patients with language barriers who are referred for either treadmill or supine bicycle stress testing may not achieve adequate workloads due to misunderstanding instructions or anxiety, even in the presence of professional interpreters.

### Congenital heart disease (pediatric patients)

The role of stress echocardiography in the pediatric population has increased over the past few decades. Children now routinely undergo cardiac stress testing for multiple reasons which include, but are not limited to, an evaluation of exercise-induced symptoms, assessment of functional capacity, assessment of the efficacy of medical and surgical interventions in patients with congenital heart disease (i.e., atrial or ventricular left to right shunts, aortic coarctation, post-anomalous coronary artery repair, post-arterial switch, congenitally corrected transposition of the great arteries, tetralogy of Fallot, hypoplastic left heart syndrome and Fontan palliation, post-cardiac transplant), prior to sports participation, and assessment for arrhythmias (i.e., long-QTc syndrome, catecholaminergic polymorphic VT, arrhythmogenic right ventricular dysplasia) (106). When feasible, exercise-based stress testing is the preferred modality of stress testing as it helps to determine functional capacity. In young children, though treadmill-based exercise can be particularly challenging, it remains the preferred modality of exercise due to the strong resemblance to normal daily activities (107). Safety precautions are important and additional stress personnel to help safeguard the child during stress testing may be necessary. Bicycle-based stress testing is also feasible, though when children are too young (<6 years of age), they may be unable to reach the pedals or handlebars; additionally, they may not know how to cycle, limiting the utility of the exam. Pharmacologic stress echocardiography may be utilized as well. Both dobutamine and vasodilatory agents can be used with similar protocols as the adult population. Though not as frequently done as compared to the adult population, pediatric stress testing appears to be generally well-tolerated. Certain higher risk phenotypes, as listed in the AHA Guidelines on Clinical Stress Testing in the Pediatric Age Group, should either be avoided or performed cautiously with a physician present (pulmonary hypertension, documented long-QTc syndrome, dilated/restrictive cardiomyopathy with heart failure or arrhythmia, history of a hemodynamically unstable arrhythmia, hypertrophic cardiomyopathy with symptoms or greater than mild left ventricular outflow tract obstruction or documented arrhythmia, greater than moderate baseline airway obstruction, routine testing in Marfan syndrome or in Marfan syndrome patients with activity-related chest pain, patients suspected to have myocardial ischemia, and unexplained exertional syncope) (106).

### Stress testing and pregnancy

Patients with known congenital or valvular disease who plan to become pregnant should be considered for an exercise stress test prior to planned pregnancy as abnormal chronotropic response in patients with known congenital heart disease has been shown to correlate with adverse pregnancy outcomes (108). In patients who are already pregnant, though strenuous exercise in pregnancy had previously been reported to lead to fetal bradycardia as a reflex vagal response to significant hypoxia from maternal hypotension and reduced uterine blood flow (109, 110), more recent studies suggest that exercise stress testing generally appears safe both for the patient and fetus. A single center study of 23 pregnant patients in late gestation (average of 35 weeks gestation, range 31 to 38 weeks gestation) subjected patients to maximal bicycle exercise stress testing (average peak heart rate 176 bpm), reporting no significant fetal heart rate change (111). Similarly, another study investigating the effects of treadmill stress testing in pregnant women between 28 and 32 weeks gestation found no significant adverse patient or fetal outcomes (112). As such, exercise stress testing can be performed safely in pregnant patients, though patient mobility and altered balance should be taken into account when selecting between a treadmill or bicycle-based (either upright or supine) modality (113). ESC guidelines for the management of cardiovascular diseases during pregnancy recommends that if a stress test is required in a pregnant patient, an exercise-based stress test is preferred with a submaximal heart rate target (80% of MPHR) (114). Dobutamine (category B in pregnancy) stress testing should generally be avoided during pregnancy when other options exist.

## Conclusion

Exercise and pharmacologic echocardiographic stress testing are central components of cardiovascular care. As such, these tests warrant careful consideration in regard to appropriate test selection, along with a thorough knowledge of potential major adverse effects and contraindications.

Exercise-based stress testing is the modality of choice, if a patient is able to exercise, as a patient's exercise capacity and symptoms can be gauged, and cardiac electromechanical response is preserved. Furthermore, exercise stress testing is safer when compared to pharmacologic modalities, with low rates of major, life-threatening patient complications. However, in light of the recent COVID-19 pandemic and the accompanying uncertainly regarding the safety of both the patients undergoing exercise-based stress testing and of the healthcare personnel conducting these tests, the number of stress echocardiograms that were performed internationally decreased by 83% at the height of the pandemic. Though there is an augmented risk of aerosol generation with exercise, when proper precautions are taken, there is minimal infectious risk to both the patient and healthcare personnel conducting the tests. Additionally, the use of facial coverings, if needed, does not significantly impact patient functional capacity or the sensitivity of ischemic detection.

Pharmacologic stress testing with both dobutamine and vasodilators is frequently utilized as well. Dobutamine-based stress testing is associated with a higher frequency of major adverse side effects due to its pro-arrhythmic risk. Vasodilators, such as dipyridamole and adenosine, can be utilized as well; however, due to cross-reactivity with multiple receptors, patients must be appropriately screened to avoid adverse effects.

Stress testing in patients with valvular dysfunction (i.e., low flow, low gradient aortic stenosis, rheumatic mitral stenosis, mitral regurgitation) represents a complex, higher-risk group of patients for which potential adverse effects of exercise and pharmacologic stress testing have not been well-delineated. In addition, special populations of patients undergoing stress testing—patients with language barriers, pediatric patients, and pregnant patients—also represent a higher risk phenotype of patient undergoing stress testing that requires close monitoring and appropriate planning.

Overall, the risk of major, life-threatening adverse patient outcomes with both exercise and pharmacologic echocardiographic stress testing is exceedingly low. However, since first utilized, these tests have been increasingly employed in more complex and higher-risk patients than the initially intended patient population (i.e., intermediate risk of obstructive coronary artery disease), and as such, the previously established risk profile may not adequately convey stress testing in a higher risk patient population. As a well-validated and frequently utilized diagnostic tool within cardiovascular medicine, continued studies and evaluation of adverse patient outcomes will continue to be important, so as to minimize the risk of future adverse outcomes to both patients and healthcare personnel.
